# Biomarkers to Guide the Timing of Surgery: Neutrophil and Monocyte L-Selectin Predict Postoperative Sepsis in Orthopaedic Trauma Patients

**DOI:** 10.3390/jcm10102207

**Published:** 2021-05-20

**Authors:** Gabrielle Daisy Briggs, Karla Lemmert, Natalie Jane Lott, Theo de Malmanche, Zsolt Janos Balogh

**Affiliations:** 1 School of Medicine and Public Health, University of Newcastle, Callaghan, NSW 2308, Australia; gabrielle.briggs@newcastle.edu.au; 2 Trauma Service, John Hunter Hospital, New Lambton Heights, NSW 2305, Australia; natalie.lott@health.nsw.gov.au; 3 Immunlogy Department, Hunter Area Pathology Service, John Hunter Hospital, New Lambton Heights, NSW 2305, Australia; karla.lemmert@health.nsw.gov.au (K.L.); theo.demalmanche@health.nsw.gov.au (T.d.M.)

**Keywords:** L-selectin, sepsis, SIRS, multiple organ failure, trauma

## Abstract

Deciding whether to delay non-lifesaving orthopaedic trauma surgery to prevent multiple organ failure (MOF) or sepsis is frequently disputed and largely based on expert opinion. We hypothesise that neutrophils and monocytes differentially express activation markers prior to patients developing these complications. Peripheral blood from 20 healthy controls and 162 patients requiring major orthopaedic intervention was collected perioperatively. Neutrophil and monocyte L-selectin, CD64, CD11, CD18, and CXCR1 expression were measured using flow cytometry. The predictive ability for MOF and sepsis was assessed using the Receiver Operating Characteristic (ROC) comparing to C-reactive protein (CRP). Neutrophil and monocyte L-selectin were significantly higher in patients who developed sepsis. Neutrophil L-selectin (AUC 0.692 [95%CI 0.574–0.810]) and monocyte L-selectin (AUC 0.761 [95%CI 0.632–0.891]) were significant predictors of sepsis and were not significantly different to CRP (AUC 0.772 [95%CI 0.650–0.853]). Monocyte L-selectin was predictive of MOF preoperatively and postoperatively (preop AUC 0.790 [95%CI 0.622–0.958]). CD64 and CRP were predictive of MOF at one-day postop (AUC 0.808 [95%CI 0.643–0.974] and AUC 0.809 [95%CI 0.662–0.956], respectively). In the perioperative period, elevated neutrophil and monocyte L-selectin are predictors of postoperative sepsis. Larger validation studies should focus on these biomarkers for deciding the timing of long bone/pelvic fracture fixation.

## 1. Introduction

Modern advances in critical care have greatly improved the survival rates of victims of severe trauma. However, for those who survive the initial injuries, multiple organ failure (MOF) remains the leading cause of late post-injury mortality [1]. The independent predictors of postinjury MOF can be grouped into patient factors (age, gender, genetic polymorphism), injury factors (injury severity, injury pattern, shock severity), and treatment factors (time to haemorrhage control, resuscitation, surgical interventions). While the patient factors and injury factors are not modifiable during trauma patient management, treatment factors are important modifiable predictors of MOF. Research into modifiable treatment factors over the past 15 years has advocated prompt haemorrhage control and largely optimised traumatic shock resuscitation. However, less attention is paid to the non-lifesaving major surgical interventions, which are essential for facilitating patient mobility, better positioning, shortening ICU stay, and minimising immobilisation associated complications. These procedures frequently involve the surgical stabilisation of long bones and the axial skeleton. The effect of these procedures’ invasiveness and timing on patient recovery is frequently disputed and largely based on expert opinion.

The decision-making around the timing of surgery is often clouded by the difficulty in predicting those who will recover from a short postoperative systemic inflammatory response (SIRS) episode and those who are at high risk of developing sepsis and MOF post-operatively. Biomarkers such as C-reactive protein and procalcitonin have been repeatedly shown as robust diagnostic markers for sepsis and septic shock. However, trauma patients represent a cohort where baseline inflammation is high, and studies of single immune biomarkers have produced conflicting results for predicting immune/infection-related complications.

Innate immune system cellular responses have been consistently shown to have a pivotal role in the pathogenesis of MOF. These involve a coordinated response of low-affinity binding to and rolling along the endothelium, followed by irreversible attachment and subsequent transmigration into tissues, all of which are regulated by dynamic cell surface protein expression and responses to chemokines and cytokines. Several studies have shown that patients with poor outcomes from traumatic injury display altered leukocyte phenotypes [2,3,4,5]; however, so far the focus has been on predicting risk in the initial period after injury. This study examines the changes in cell surface markers of neutrophil and monocyte activation, namely, L-selectin (CD62L), CD64, CD11b, CD18, and CXCR1 in the perioperative period to definitive orthopaedic surgery, to predict poor outcomes of postoperative sepsis and MOF at a clinically meaningful time point for decision making about planned urgent surgery. We hypothesise that at the time of their definitive orthopaedic trauma surgery, major trauma patients who develop postoperative complications will have altered cell surface marker expression.

## 2. Materials and Methods

### 2.1. Patient Selection

This study was approved by the Hunter New England Health Human Research Ethics Committee (10/11/17/4.05) and included a convenience sample (secondary surgery performed during office hours on workdays) of trauma patients presenting to the John Hunter Hospital between October 2011 and February 2015 with pelvis, femoral, tibial, and acetabular fractures requiring open reduction and internal fixation. Exclusion criteria were patients <16 years of age, patients who developed sepsis/MOF prior to surgery, or more than 6 weeks post-operatively. Healthy controls were also included in the study. A preoperative EDTA peripheral blood sample from patients approximately 30 min prior to surgery for flow cytometry measurements. In addition, postoperative samples of 30 min, 7 h, 1 day, and 3 days after surgery were collected to determine whether postoperative profiling of activation markers was also of use for early prediction of postoperative outcomes. Patient demographics, routine pathology test results, and additional data for SIRS/MOF/sepsis criteria were recorded prospectively. SIRS was defined using established SIRS criteria, sepsis was defined as SIRS with positive blood culture and or proven source of infection based on positive cultures/clinical signs. MOF was defined as a Denver Score >3 after the first 48 h post-injury. C-reactive protein (CRP) was measured at the same time as blood collection for flow cytometry for comparators as commonly used markers of infection/inflammation.

### 2.2. Flow Cytometry

Immediately after blood collection, 100ul of EDTA whole blood was labelled with CD45-ECD, CD18-FITC, CD62L-PE (Beckman), and CD181-PC5 (BD Biosciences) or with CD45-ECD, CD64-FITC, and CD11b-PE for 10 min at room temperature,. Samples underwent red cell lysis and fixation using the ImmunoPrepTM reagent system (Beckman-Coulter) according to the manufacturer’s instructions and were analysed using a 3 laser Beckman-Coulter Navios Flow Cytometer. Cell surface marker expression was measured by gating on CD45+ granulocyte and monocyte populations and collecting the mean fluorescence intensity (MFI) values for the CD18, CD62L, CD181, CD64, and CD11b markers on granulocytes and the CD62L, CD64, and CD11b markers on monocytes. 

### 2.3. Data Analysis

Patients were sub grouped into 4 groups: SIRS, sepsis, MOF, and non-complicated trauma. The sepsis group included patients who developed sepsis postoperatively, the MOF group included patients who developed MOF postoperatively. The SIRS group included patients who developed postoperative SIRS, who did not develop sepsis or MOF. The non-complicated trauma group included all other patients in the study who did not develop SIRS, sepsis, or MOF postoperatively. Patients who had post-injury SIRS that resolved prior to surgery were also included in this non-complicated group. Demographics were compared among groups using a Kruskal-Wallis one-way analysis of variance by ranks with Dunn’s multiple comparison test for post-hoc analysis. 

Since original values were in the form of MFI, these were converted to a fold increase from the median healthy control MFI for each marker. Natural history of cell surface markers was compared among groups using a mixed-effects analysis on log-transformed values with Tukey’s post-hoc multiple comparisons test to examine the effect of both time and outcome, and healthy control values were compared to timepoints in clinical subgroups using a Mann-Whitney Test.

Denver MOF score and sepsis/SIRS criteria were used to determine the onset of complications and any cell surface marker data points after this time were omitted from the analysis. Receiver-Operating Characteristic (ROC) analyses were performed to test the predictive ability of the cell surface markers for MOF and sepsis. Different groupings of the data were tested to represent clinically relevant scenarios in the perioperative period, namely, MOF versus no MOF, sepsis versus no sepsis, sepsis versus SIRS, and MOF versus sepsis/SIRS. Predictive accuracy was measured as the area under the curve and Youden’s index was used to determine cut-off values. Cut-off values were represented as fold-increased from healthy controls rather than MFI. Pairwise comparisons between ROC curves were performed using the DeLong method. 

Multiple logistic regression analysis was used to determine whether cell surface biomarkers were dependent on known predictors of MOF and sepsis, and included covariates of age, sex, ISS, time from injury, and shock severity (admission base deficit). Significant results from ROC analyses were used for the regression model and markers (cell surface markers and CRP) were added to the model individually rather than in combination with each other. Markers that were found to be independently associated with sepsis and MOF were then tested in combination in the regression analysis. Two-tailed *p* values are reported with significance defined as *p* < 0.05. Statistical analysis and figure generation used GraphPad Prism 8 software and MedCalc v 19.7.1.

## 3. Results

### 3.1. Patient Demographics

Demographics of the 162 trauma patients recruited into the study are shown in Table 1. One patient was excluded due to developing sepsis preoperatively, while another patient was excluded due to developing MOF preoperatively. There was only one in-hospital death and this patient was in the SIRS group. Blood was also collected from 20 healthy controls throughout the study (mean age 52 ± 18, 55% male). Meanwhile, 30 patients developed postoperative SIRS without sepsis (19%), nineteen patients developed postoperative sepsis (12%), six patients developed postoperative MOF (3.7%), and five of the six patients with postoperative MOF also had sepsis. Patients with postoperative SIRS met the criteria at a median of a 1-day postop (range 1–10 days), while patients who went on to develop sepsis also had SIRS at a median of 1-day postop (range 1–4 days), but confirmed infection at a median of 5 days postop (range 1–24 days). All patients who developed postoperative MOF had postoperative SIRS at day one postop and met MOF criteria at a median of 1-day postop (range 1–5 days). There were no significant differences in the patient age or gender ratios among the different groups; however, the healthy control group had significantly more females than the non-complicated trauma (*p* = 0.04) and sepsis subgroups (*p* = 0.001). ISS was significantly higher in the sepsis, MOF, and SIRS groups compared to the non-complicated trauma group. However, ISS did not differ when the sepsis, MOF, and SIRS groups were compared to each other. Admission base deficit was also significantly greater in the sepsis group compared to the non-complicated group (*p* < 0.05); however, there were no significant differences when the sepsis, MOF, and SIRS groups were compared to each other. The time from injury to surgery ranged from 30 min to 10 days; however, there were no significant differences among groups in the timing of surgery and immediate preoperative vital signs and laboratory variables. 

### 3.2. Natural History of Perioperative Neutrophil and Monocyte Cell Surface Marker Expression

**Figure 1:**
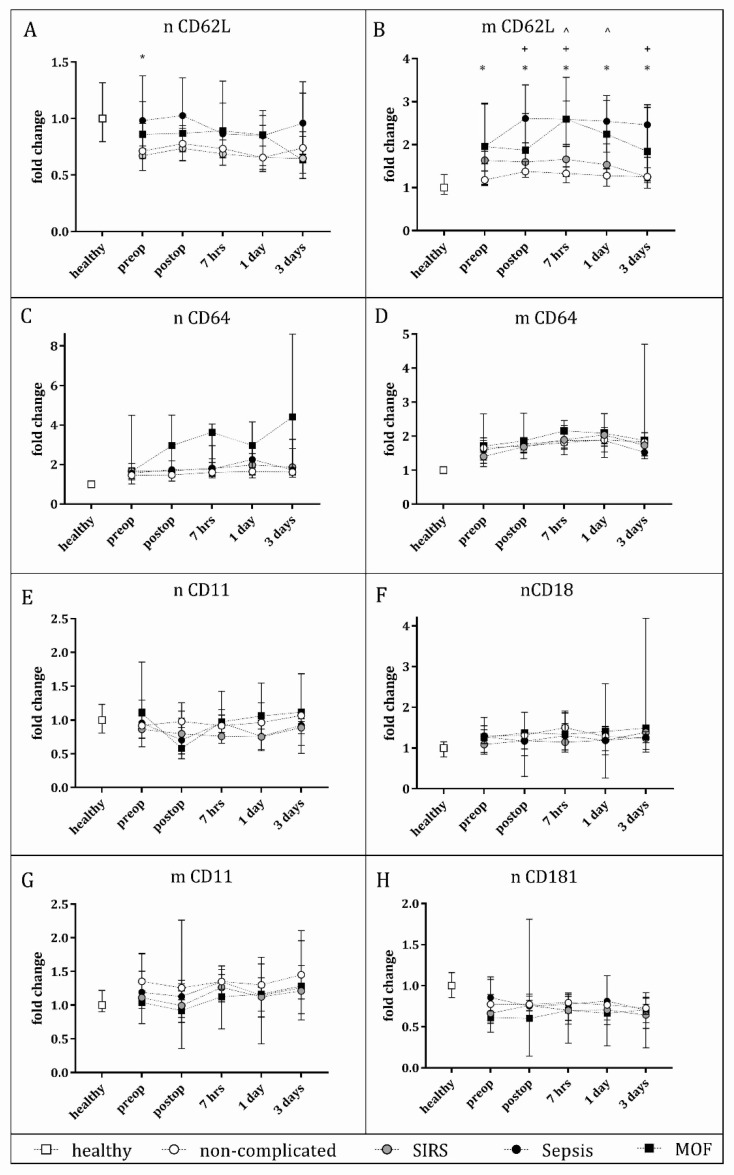
Neutrophil (n) and monocyte (m) expression of activation markers over the perioperative period. Peripheral blood was collected preoperatively to 3 days postoperatively from trauma patients and cell surface marker expression analysed using flow cytometry. CD45+ granulocyte and monocyte populations were gated and mean fluorescence intensity values (MFI) for each marker were collected and grouped by outcome along with healthy controls. Cell surface markers analysed included (**A**) neutrophil L-selectin (nCD62L), (**B**) monocyte L-selectin (mCD62L), (**C**) neutrophil CD64, (**D**) monocyte CD64, (**E**) neutrophil CD11, (**F**) neutrophil CD18, (**G**) monocyte CD11, (**H**) neutrophil CD181. Plotted values represent fold change over the median healthy control values. Significant differences are shown as * = sepsis vs. non-complicated trauma, + sepsis vs. SIRS, and ^ MOF vs. non-complicated trauma. * = *p* < 0.05, ** = *p* < 0.01, *** = *p* < 0.001. Significant differences between healthy controls and clinical groups and significant effects between timepoint have been omitted from the graph for simplicity and are available in Supplementary Table S1.

The expression of cell surface markers on neutrophils and monocytes relative to healthy controls over the perioperative period are shown in Figure 1. Neutrophil L-selectin (nCD62L) expression in the sepsis and MOF groups was not significantly different from that of healthy controls. However, both the non-complicated and SIRS groups had significantly lower neutrophil L-selectin at all perioperative time points, and at the preop, 7 h, and 3 day time points, respectively, compared to healthy controls (Supplementary Table S1). Mixed-effects analysis indicated no significant variation of neutrophil L-selectin over time; however, there was significant variation among clinical subgroups (*p =* 0.017). Post-hoc analysis found that sepsis patients had significantly higher neutrophil L-selectin compared to the non-complicated patients at the preoperative time point only (Figure 1A). 

Monocyte L-selectin expression (mCD62L Figure 1B) was found to be significantly elevated from that of healthy controls in the sepsis, MOF, and SIRS groups but not the non-complicated group, with the sepsis group showing significantly higher expression at all time points, the MOF group at all but the 3-day time point, and the SIRS group at the postop, 7 h, and 1-day time points. In comparing clinical groups to each other, monocyte L-selectin was found to vary significantly with both time (*p* = 0.006) and by outcome (*p* < 0.0001). Those who went on to develop sepsis had significantly higher monocyte L-selectin compared to non-complicated patients at all time points, and significantly higher than patients with SIRS at the postop, 7 h, and 3-day time point. Sepsis and MOF patients did not significantly differ from each other in monocyte L-selectin, and MOF patients were found to have higher monocyte L-selectin compared to the non-complicated group at the 7 h and 1-day time points only. SIRS patients’ monocyte L-selectin did not differ from that of non-complicated patients at any time point. Post-hoc analysis of the variation over time indicated that in the non-complicated patients only, the postop and 7 h monocyte L-selectin was significantly higher than the preop value.

While the median neutrophil CD64 expression was up to 4.4-fold higher in MOF patients than in other groups at the postoperative time points, this was not significant and no other significant differences in neutrophil CD64 expression were evident among any of the clinical groups across the perioperative period (Figure 1C). However, neutrophil CD64 expression was significantly higher than healthy controls in all clinical groups at all time points. Monocyte CD64 expression did not differ among subgroups; however, there was significant variation with time (*p* = 0.0016), with the sepsis group expressing significantly higher CD64 at the 7 h time point compared to the preop value, and at the 7 hours and day 1 timepoint compared to the preop value in the SIRS and non-complicated groups.

The expression of the integrins, CD11, and CD18 (Figure 1E–F) on neutrophils and CD11 on monocytes (Figure 1G) did not significantly differ between any of the clinical groups. However, there was a significant effect of time (*p* 0.015 and 0.017 and 0.039, respectively), with the postop monocyte CD11 being significantly decreased from that of the 7 h time point in the SIRS and non-complicated groups. Small but significant increases and decreases in CD11 and CD18 expression were evident between healthy controls and other time points in clinical groups. However, due to the fluctuation in these markers over time, there was no consistent trend observed. 

Neutrophil CXCR1 expression (Figure 1H) did not differ among clinical groups and no significant effect of time was evident. Compared to healthy controls, all trauma patient groups had significantly lower neutrophil CXCR1, apart from sepsis patients at day 1 and MOF patients at the postop timepoint. 

### 3.3. ROC Analyses

ROC analyses were performed to determine the predictive ability of the cell surface markers and CRP for sepsis and MOF, with significant AUCs and best cut-off values shown in Table 2, Table 3 and Table 4. Overall, neutrophil and monocyte L-selectin were significantly predictive for sepsis and MOF with comparable AUCs to CRP, while neutrophil CD64 was predictive of MOF, but not sepsis. 

At the preoperative time point, the predictors of sepsis were monocyte L-selectin, with an AUC of 0.761 (95%CI 0.632–0.891) *p* = 0.0003, neutrophil L-selectin with an AUC of 0.692 (95%CI 0.574–0.810) *p* = 0.007, and CRP with an AUC of 0.772 (95%CI 0.650–0.893) *p* = 0.0001. However, AUCs were not significantly different from one another. Given that the sepsis, MOF, and SIRS patients were more severely injured compared to the non-complicated patients. We also tested the ability for cell surface markers and CRP to predict MOF and sepsis within the complicated patient groups alone, which is likely to represent a realistic target patient group in whom clinicians would want to assess the risk of sepsis/MOF (e.g., SIRS vs. sepsis in the ICU). CRP, monocyte and neutrophil L-selectin were again the only three significant predictors of sepsis, with respective AUCs of 0.79 2 (95%CI 0.657–0.928) *p* = 0.0007, 0.720 (95%CI 0.564–0.876) *p* = 0.011 and 0.731 (95%CI 0.589–0.873) *p* = 0.008, which were not significantly different from one another.

In addition to the preop timepoint, monocyte L-selectin was also predictive of sepsis at all postoperative time points in the whole cohort (Table 2) and within the complicated cohort (Table 3), with the highest AUC at the immediate postoperative timepoint. Neutrophil L-selectin was also predictive of sepsis postoperatively in the whole cohort at the postop and the day 1 time point, and within the complicated cohort at the postop timepoint only. CRP was predictive of sepsis at 1 and 3 days postop; however, it was no longer predictive of sepsis at day 3 when applied to the complicated patients only. In comparing AUCs, the only significant differences were between monocyte L-selectin and neutrophil L-selectin at the postop (*p* = 0.0006) and 7 h (*p* = 0.012) timepoints in the whole cohort.

Preoperatively, the only significant predictor of MOF was monocyte L-selectin, with an AUC of 0.790 (95%CI 0.622–0.958) *p* = 0.017. However, when this was restricted to complicated patients, no significant result was found. Postoperatively, monocyte L-selectin was predictive of MOF at the 7 h and 1-day timepoint. CD64 showed a high predictive ability for MOF at the postop, 7 h, and 1 day time points, while CRP was predictive for MOF at the day 1 and 3 time points. There were no significant differences between AUCs for monocyte L-selectin, neutrophil CD64, and CRP. When ROC analyses were performed on the complicated patients only (data not shown in table), CD64 could predict MOF at the 7 h (AUC 0.845 [95%CI 0.774–0.923] *p* = 0.011) and 1-day timepoint (AUC 0.754 [95%CI 0.640–0.974] *p* = 0.011). However, no other significant values were evident for other markers.

### 3.4. Multivariate Logistic Analysis

Given that trauma patient outcomes can be driven by a myriad of factors, we used multivariate logistic regression to determine whether L-selectin and CD64 were independently associated with sepsis and MOF, when age, sex, ISS, admission base deficit, and the time from injury were included in the regression model. Regression models were generated for each perioperative timepoint. Monocyte and neutrophil L-selectin were both independently associated with sepsis at all time points except for day 1 for neutrophil L-selectin (preop results shown in Table 5, all timepoints shown in Table S2). However, CRP was found not to be independently associated with sepsis. ISS and the time from injury were consistently associated with sepsis in the regression models. Age, sex, and admission BD were not significant. When neutrophil and monocyte L-selectin were combined at each timepoint, monocyte L-selectin remained independently associated with sepsis, while neutrophil L-selectin did not. 

While monocyte L-selectin and CD64 were shown to be predictors of MOF in ROC analyses, multivariate logistic regression indicated that these were not independently associated with MOF at any time point (data not shown), with ISS being the only covariate that was independently associated with postoperative MOF (OR 1.14 95%CI 1.05–1.29).

## 4. Discussion

This study has provided a clinically focused update on the perioperative expression of innate immune cell activation markers, demonstrating that trauma patients’ neutrophils and monocytes display differential expression of activation markers prior to the development of sepsis, MOF, and SIRS. Given the current inability to predict which trauma patients are at risk of developing sepsis or MOF after major musculoskeletal surgery, these findings identify key markers that may identify at-risk patients for whom surgery should be delayed. Additionally, since the pathobiological mechanisms underlying sepsis and MOF development are not well understood, this study sheds some light on potential differences in the cellular responses to infection and inflammation. 

The major finding in this study was that L-selectin expression on monocytes and to a lesser extent, neutrophils, is increased preoperatively in patients who later developed sepsis. This was also evident at postoperative time points, indicating that monocyte and neutrophil L-selectin could be used for monitoring patients for early sepsis. Postoperative MOF could also be predicted using monocyte L-selectin; however, with only six patients developing MOF and five of the six also developing sepsis, this would need to be re-evaluated with larger sample sizes.

L-selectin is a constitutively expressed adhesion molecule on the surface of leukocytes that mediates low-affinity binding and rolling along the endothelium. Decreases in L-selectin expression are considered to be indicative of leukocyte activation, as L-selectin shedding occurs as cells transmigrate into tissues. Given this interpretation, the fact that sepsis patients have higher L-selectin on both neutrophils and monocytes suggests that they have a higher capacity for leukocyte margination. However, they have proportionally fewer leukocytes entering tissues, which may predispose them to the spread of infection. Alternatively, increased leukocyte output from the bone marrow may also account for an increased proportion of cells with high L-selectin. 

Elegant studies from 1998–2001 described the natural history of L-selectin expression on leukocytes in trauma patients, showing an initial increase in expression in the hours following injury [5,6] and a return to healthy levels in the 24–48 h period [4]. Given that the time course studied herein is the period surrounding major orthopaedic interventions, we have captured a later window where injury-induced acute responses are on the decline or have returned to normal, reflecting data from longer periods as shown in the study of Seekamp et al. [4]. The finding that neutrophil and monocyte L-selectin were associated with sepsis independently of injury severity and other known predictors indicates that increased L-selectin is also being driven by other factors. Previous interventions, resuscitation, medication, undetectable infections, and sterile hyper-inflammation are other potential drivers of cell surface marker expression, all of which can occur prior to non-lifesaving orthopaedic surgery. 

Two prior studies of post-injury L-selectin expression conclude that they could not identify any changes in L-selectin that would predict post-injury complications such as MOF [5,6], while others reported decreased granulocyte (4) and increased monocyte [3] L-selectin in MOF patients but did not investigate the trends in sepsis patients. More recently, neutrophil L-selectin measured on admission using a point-of-care flow cytometry-based assay was shown to correlate with infectious complications in polytrauma patients [7]. Our data suggest that for the timing of orthopaedic surgery, monocyte l-selectin should also be investigated as a predictor of infectious complications.

L-selectin has been studied intensively in its soluble form for its potential as a biomarker in predicting outcomes from trauma [8]. Once shed from the leukocyte surface during activation, soluble L-selectin (sL-selectin) accumulates in the circulation and can be measured in serum/plasma using ELISA. Several studies have made associations with sL-selectin and outcomes in trauma, and the most compelling evidence seems to be that sL-selectin is elevated in sepsis, but decreased in those who develop ARDS [8,9]. However, the predictive ability of this marker is still considered to be poor and some argue its usefulness as a biomarker is complicated by dilution due to resuscitation fluids and blood products. One limitation of the current study is a lack of complementary sL-selectin measurements; however, this study was focused on assessing cell surface biomarkers rather than mechanisms underlying these processes. Furthermore, sL-selectin reflects the total neutrophil, monocyte, and lymphocyte shedding at any given time, rather than allow the time-resolved profiling of expression by cell type.

Biomarkers associated with the innate immune system, especially soluble biomarkers such as cytokines IL-6 and IL-10, CRP, and procalcitonin have shown variable ability to identify hyper inflammation and/or sepsis [10]. In the context of post-injury sepsis, inflammation/immune-related biomarkers may become non-specific due to the much higher baseline inflammation in these patients, so targeted studies of trauma patients are essential. Of these innate immune markers, procalcitonin has been shown to have the ability to differentiate sepsis patients within an injured, critically ill cohort [11]; however, it is not known whether this could be used to determine the risk of sepsis preoperative to orthopaedic surgery. CRP is currently used in trauma patients as a gauge for inflammation/infection, but it is understood from the literature [10] and at the bedside that this is non-specific in the trauma cohort, who experience elevated CRP from both injury and multiple surgeries. In the present study, we found that high CRP was associated with sepsis; however, this association was dependent on injury severity/patient factors.

Neutrophil CD64 was included in this study as it is a known marker of neutrophil activation and is also a strong candidate for diagnosing sepsis, outperforming CRP and procalcitonin [12]. We did not find any association with post-injury sepsis and neutrophil CD64. It is worth noting that in this study, CD64 was markedly increased from healthy controls in all trauma patients, suggesting that post-injury increases in CD64 mask any additional increases due to sepsis, and therefore, CD64 may have low specificity in trauma patients. Due to its success in sepsis diagnosis, point-of-care tests for CD64 are currently in development, so trauma patients must be specifically studied further to determine the diagnostic utility of CD64 in this cohort.

This study was not without its limitations. Since the population studied consisted of trauma patients who survived the initial injuries to go on to have non-lifesaving orthopaedic surgery, the rate of MOF and sepsis was low, so our results should be interpreted with caution. Importantly, the patients included here are those who were not complicated at the preoperative time point, i.e., they did not have existing MOF/sepsis and were deemed safe for major surgery, so the complicated patients in our study are likely to be those for whom the risk was truly modifiable by delaying surgery or through other interventions. Inclusion of other complications known to exist in trauma patients, especially in the elderly, such as venous thromboembolism, myocardial infarction, and stroke may have provided further insight into the perioperative changes in innate immune cell surface markers. However, they were not collected as part of this investigation. Unfortunately, we did not measure procalcitonin as a comparator as this was not routinely collected on suspected sepsis patients at our institution. This would have allowed us to better understand how monocyte-L-selectin measures up against the current state-of-the-art. Another limitation is that we did not collect SOFA scores in this study, as at the time of its design, the sepsis definition did not use SOFA. Rather, the Denver MOF score was used for MOF, which is known to capture more severe MOF than SOFA and is more appropriate for understanding MOF in trauma patients. The use of SOFA in this study may have further stratified the sepsis cohort by the presence/absence of organ failure and perhaps markers such as CD64 would have shown more significant results.

In conclusion, increased monocyte L-selectin and to a lesser extent, neutrophil L-selectin expression is associated with postoperative sepsis in orthopaedic trauma. Importantly, the preoperative monocyte L-selectin could be used to predict sepsis, which could not only guide the timing of surgery to prevent postoperative sepsis and resultant MOF but also prevent bone and joint infections by delaying the implantation of hardware in patients at risk of bacteraemia that could seed the implant. While neutrophil CD64 is the leading cell surface marker for sepsis diagnosis in all-comers, trauma-specific studies of these markers in the prediction of postoperative sepsis and MOF are essential to be able to better prevent poor outcomes in these at-risk patients.

## Figures and Tables

**Table 1:** Patient demographics.

Group	Healthy	Non-Complicated	SIRS	Sepsis	MOF	*p*-Value
n	20	124	30	19	6	
Age (years)	52 (18)	44 (19)	40 (18)	49 (15)	53 (18)	0.104
% Male	55 (51) * ^^	77 (42)	67 (48)	95 (23)	83 (41)	0.010
ISS		13 (13)	22 (24) ***	38 (23) ****	38 (13) ***	<0.0001
Admission base deficit (mEq/L)		−1.01 (3.12)	−2.54 (3.98)	−4.58 (3.97) *	−3.88 (5.38)	0.003
Hours to surgery		24 (60)	20 (39)	44 (114)	44 (45)	0.186
Days to MOF		0	0	0	1 (1–5)	
Days to sepsis		0	0	5 (1–24)	5 (2–7)	
Days to SIRS		0	1 (1–10)	1 (1–4)	1 (1–2)	

Data are shown as mean ± SD, apart from ISS and time to surgery, shown as median ±IQR and time to MOF, sepsis, and SIRS, shown as median with range. Significant differences to non-complicated trauma according to post-hoc analysis are indicated as follows: * = *p* < 0.05, *** = *p* < 0.001, **** = *p* < 0.0001. Significant differences to sepsis according to post-hoc analysis are indicated as ^ = *p* < 0.05 and ^^ = *p* < 0.01.

**Table 2:** Prediction of sepsis in trauma patients using ROC analysis.

	CRP AUC (95%CI)	Cut-Off	mCD62L AUC (95%CI)	Cut-Off	nCD62L AUC (95%CI)	Cut-Off
Pre	0.772 (0.650–0.893) ***	>114	0.761 (0.632–0.891) ***	>1.77	0.692 (0.574–0.810) **	>0.61
Post	-		0.821 (0.736–0.906) ****	>1.75	0.646 (0.514–0.778) *	>0.63
7 h	-		0.763 (0.634–0.892) ***	>1.89	0.631 (0.490–0.772)	
1 day	0.783 (0.682–0.884) ****	>163	0.778 (0.677–0.899) ***	>2.00	0.689 (0.580–0.814) **	>0.63
3 days	0.742 (0.612–0.873) **	>151	0.734 (0.559–0.907 **	>2.44	0.610 (0.435–0.786)	

Cut off values for CRP are in mg/L and monocyte L-selectin (mCD62L) and neutrophil L-selectin (nCD62L) cut off values are expressed as fold change from healthy control. * = *p* < 0.05, ** = *p* < 0.01, *** = *p* < 0.001, **** = *p* < 0.0001.

**Table 3:** Prediction of sepsis in complicated trauma patients.

	CRP AUC (95%CI)	Cut-Off	mCD62L AUC (95%CI)	Cut-Off	nCD62L AUC (95%CI)	Cut-Off
Pre	0.792 (0.657–0.928) ***	>107	0.720 (0.564–0.876) *	>1.94	0.731 (0.589–0.873) **	>0.61
Post	-		0.788 (0.659–0.917) **	>1.63	0.689 (0.535–0.842) *	>0.98
7 h	-		0.756 (0.601–0.912) **	>1.89	0.657 (0.493–0.821)	
1 day	0.668 (0.525–0.850) *	>159	0.702 (0.545–0.860) *	>2.00	0.651 (0.491–0.810)	
3 days	0.607 (0.498–0.794)		0.744 (0.564–0.923) *	>2.41	0.699 (0.516–0.881)	

Cut off values for CRP are in mg/L and monocyte L-selectin (mCD62L) and neutrophil L-selectin (nCD62L) cut off values are expressed as fold change from healthy control. * = *p* < 0.05, ** = *p* < 0.01, *** = *p* < 0.001.

**Table 4:** Prediction of MOF in trauma patients.

	CRP AUC (95%CI)	Cut-Off	mCD62L AUC (95%CI)	Cut-Off	nCD64 AUC (95%CI)	Cut-Off
Pre	0.709 (0.492–0.927)		0.790 (0.622–0.958) *	>1.89	0.558 (0.286–0.830)	
Post	-		0.602 (0.452–0.751)		0.770 (0.496–1.00) *	>2.16
7 h	-		0.791 (0.678–0.904) *	>1.96	0.873 (0.773–0.973) *	>2.10
1 day	0.809 (0.662–0.956) *	>158	0.792 (0.708–0.876) *	>1.99	0.808 (0.643–0.974) *	>2.50
3 days	0.811 (0.738–0.883) *	>156	0.610 (0.325–0.895)		0.726 (0.496–0.957)	

Cut off values for CRP are in mg/L and monocyte L-selectin (mCD62L) and neutrophil CD64 (nCD64) cut off values are expressed as fold change from healthy control. * = *p* < 0.05.

**Table 5:** Multivariate logistic regression for sepsis prediction.

Variable	OR (95% CI)	*p* Value
Age	0.98 (0.93–1.30)	0.510
Sex	0.13 (0.01–0.99)	0.092
ISS	1.15 (1.08–1.26)	0.0003
time since injury	1.01 (1.00–1.02)	0.026
admission base deficit	0.92 (0.75–1.11)	0.410
monocyte L-selectin	1.50 (1.21–1.98)	0.001
neutrophil L-selectin	1.56 (1.16–2.19)	0.005
C-reactive Protein	1.01 (0.99–1.01)	0.095

Preoperative ORs for monocyte and neutrophil L-selectin are shown. ORs for other variables are from the regression model using monocyte L-selectin.

## Data Availability

Data is contained within this article and supplementary material.

## Supplementary Materials

The following are available online at https://www.mdpi.com/article/10.3390/jcm10102207/s1, Table S1, Significant differences in cell surface marker expression expressed as fold change of healthy control median MFI. Table S2, Multivariate regression results for postop sepsis using neutrophil l-selectin, monocyte l-selectin or CRP at all perioperative timepoints.
