# Supplementary material for: Biomarkers to Guide the Timing of Surgery: Neutrophil and Monocyte L-Selectin Predict Postoperative Sepsis in Orthopaedic Trauma Patients
Source: J Clin Med. 2021 May 20;10(10):2207. doi: 10.3390/jcm10102207 (PMC8160833; doi:10.3390/jcm10102207)
Supplement: Supplementary file 1 [file jcm-10-02207-s001.zip › jcm-1193922supple.pdf]

Supplementary material

Table S1. Significant differences in cell surface marker expression expressed as fold change of healthy control median MFI

**n 62L**	preop	postop	7hrs	d1	d3
non-complicated	0.71**	0.78*	0.73**	0.65***	0.74*
SIRS	0.67*	0.74*	0.69**	0.65*	0.65**
sepsis	0.98	1.02	0.91	0.85	0.96
MOF	0.86	0.87	0.86	0.85	0.63

**m CD62L**	preop	postop	7hrs	d1	d3
non-complicated	1.18	1.37	1.33	1.28	1.25
SIRS	1.63	1.60**	1.66*	1.53*	1.24
sepsis	1.95**	2.61****	2.59***	2.54***	2.46***
MOF	1.95*	1.87**	2.59**	2.24**	1.84

**n CD64**	preop	postop	7hrs	d1	d3
non-complicated	1.46****	1.47****	1.68****	1.64****	1.62****
SIRS	1.67****	1.68****	1.80****	1.97****	1.87****
sepsis	1.56****	1.74****	1.77****	2.25****	1.72***
MOF	1.64**	2.95***	3.63***	2.26****	4.40***

**m CD64**	preop	postop	7hrs	d1	d3
non-complicated	1.65****	1.71****	1.81****	1.89****	1.83****
SIRS	1.40****	1.68****	1.89****	2.04****	1.73***
sepsis	1.59****	1.76****	1.87****	1.87****	1.52****
MOF	1.71***	1.86****	2.16***	2.08****	1.88***

**n CD11**	preop	postop	7hrs	d1	d3
non-complicated	0.92	0.98	0.92	0.96	1.07
SIRS	0.86	0.80*	0.76*	0.75*	0.89
sepsis	0.96	0.70*	0.96	0.76	0.92
MOF	1.10	0.58	0.98	1.06	1.12

**n CD18**	preop	postop	7hrs	d1	d3
non-complicated	1.29**	1.32**	1.50***	1.29**	1.25*
SIRS	1.09	1.18	1.15	1.19	1.39*
sepsis	1.28*	1.17	1.30*	1.19	1.27
MOF	1.26	1.38	1.34*	1.38	1.49*

m CD11	preop	postop	7hrs	d1	d3
non-complicated	1.35*	1.26	1.35**	1.30*	1.45**
SIRS	1.11	0.99	1.26	1.12	1.21
sepsis	1.19	1.13	1.35*	1.15	1.26**
MOF	1.04	0.92	1.12	1.16	1.28

n CD181	preop	postop	7hrs	d1	d3
non-complicated	0.77**	0.77**	0.79**	0.77**	0.73**
SIRS	0.66**	0.76***	0.70****	0.70****	0.64***
sepsis	0.76	0.75**	0.77**	0.80	0.70**
MOF	0.61*	0.60	0.70*	0.66*	0.71*

Significant differences between healthy controls and clinical subgroups are denoted by, *=p<0.05, ** = p<0.01, ***p<0.001, **** = p<0.0001.

Table S2. Multivariate regression results for postop sepsis using neutrophil l-selectin, monocyte l-selectin or CRP at all perioperative timepoints.

Variable	OR	95% CI	P value
Age	0.98	0.9398 to 1.026	0.4551
Sex	0.22	0.01039 to 1.515	0.1879
ISS	1.16	1.089 to 1.275	<0.0001
time from injury	1.01	0.9973 to 1.019	0.1153
admission bd	0.96	0.8069 to 1.131	0.6204
neutrophil l-selectin preop	1.56	1.159 to 2.190	0.0051

Variable	OR	95% CI	P value
Age	0.99	0.9542 to 1.034	0.7686
Sex	0.27	0.01361 to 1.719	0.2403
ISS	1.15	1.078 to 1.243	0.0001
time from injury	1.01	1.001 to 1.020	0.0248
admission bd	0.99	0.8354 to 1.174	0.9234
neutrophil l-selectin postop	1.27	1.003 to 1.624	0.0486

Variable	OR	95% CI	P value

Age	1.00	0.9567 to 1.038	0.8928
Sex	0.22	0.008148 to 1.612	0.2179
ISS	1.14	1.073 to 1.241	0.0002
time from injury	1.01	1.001 to 1.021	0.0234
admission bd	0.99	0.8318 to 1.164	0.8594
neutrophil l-selectin 7hrs	1.38	1.066 to 1.789	0.0109

**Variable**	**OR**	**95% CI**	**P value**
Age	0.99	0.9544 to 1.033	0.7578
Sex	0.23	0.01133 to 1.496	0.1948
ISS	1.15	1.076 to 1.240	0.0001
time from injury	1.01	0.9991 to 1.019	0.0556
admission bd	0.96	0.8144 to 1.136	0.6637
neutrophil l-selectin 1day	1.24	0.9334 to 1.583	0.1073

**Variable**	**OR**	**95% CI**	**P value**
Age	0.99	0.9415 to 1.034	0.62
Sex	0.39	0.01896 to 2.786	0.4122
ISS	1.17	1.090 to 1.294	0.0002
time from injury	1.01	0.9998 to 1.022	0.046
admission bd	1.00	0.8284 to 1.207	0.9974
neutrophil l-selectin 3 days	1.48	1.143 to 2.030	0.0058

**Variable**	**OR**	**95% CI**	**P value**
Age	0.98	0.9380 to 1.030	0.5074
Sex	0.13	0.005801 to 0.9879	0.0921
ISS	1.15	1.077 to 1.263	0.0003
time from injury	1.01	1.001 to 1.023	0.0264
admission bd	0.92	0.7491 to 1.112	0.4085
monocyte l-selectin preop	1.50	1.207 to 1.979	0.001

**Variable**	**OR**	**95% CI**	**P value**
Age	1.00	0.9530 to 1.047	0.9986

Sex	0.30	0.01460 to 2.199	0.3027
ISS	1.16	1.084 to 1.276	0.0002
time from injury	1.01	1.004 to 1.026	0.0081
admission bd	1.05	0.8582 to 1.282	0.655
monocyte l-selectin post	1.50	1.219 to 1.994	0.0009

**Variable**	**OR**	**95% CI**	**P value**
Age	0.99	0.9446 to 1.035	0.6782
Sex	0.53	0.02583 to 3.849	0.5784
ISS	1.15	1.071 to 1.249	0.0004
time from injury	1.01	1.001 to 1.022	0.0244
admission bd	1.06	0.8729 to 1.305	0.5532
monocyte l-selectin 7hrs	1.45	1.181 to 1.865	0.0013

**Variable**	**OR**	**95% CI**	**P value**
Age	0.99	0.9458 to 1.034	0.6642
Sex	0.39	0.01849 to 2.996	0.431
ISS	1.15	1.075 to 1.265	0.0005
time from injury	1.01	0.9950 to 1.018	0.184
admission bd	0.97	0.8102 to 1.167	0.778
monocyte l-selectin 1 day	1.27	1.071 to 1.555	0.0107

**Variable**	**OR**	**95% CI**	**P value**
Age	0.98	0.9251 to 1.032	0.4739
Sex	0.23	0.008433 to 2.061	0.2575
ISS	1.19	1.097 to 1.348	0.0005
time from injury	1.01	0.9926 to 1.018	0.3217
admission bd	1.20	0.9417 to 1.633	0.1762
monocyte l-selectin 3 day	1.57	1.211 to 2.253	0.0032

**Variable**	**OR**	**95% CI**	**P value**
Age	1.00	0.9566 to 1.035	0.8302

Sex	0.24	0.01229 to 1.537	0.2047
ISS	1.14	1.071 to 1.229	0.0002
time from injury	1.01	0.9985 to 1.018	0.0726
admission bd	1.01	0.8392 to 1.202	0.9515
CRP pre	1.01	0.9989 to 1.014	0.0949

**Variable**	**OR**	**95% CI**	**P value**
Age	1.01	0.9616 to 1.049	0.8359
Sex	0.37	0.01797 to 2.656	0.3921
ISS	1.15	1.079 to 1.267	0.0003
time from injury	1.01	0.9981 to 1.020	0.0799
admission bd	1.00	0.8273 to 1.213	0.9728
CRP 1 day	1.01	0.9994 to 1.015	0.0593

**Variable**	**OR**	**95% CI**	**P value**
Age	1.00	0.9586 to 1.037	0.9134
Sex	0.23	0.01178 to 1.493	0.195
ISS	1.14	1.075 to 1.234	0.0001
time from injury	1.01	0.9989 to 1.018	0.0629
admission bd	1.00	0.8316 to 1.190	0.9814
CRP 3 days	1.01	0.9972 to 1.017	0.1398
